# Bilobalide modulates serotonin-controlled behaviors in the nematode *Caenorhabditis elegans*

**DOI:** 10.1186/1471-2202-10-62

**Published:** 2009-06-22

**Authors:** Marishka K Brown, Yuan Luo

**Affiliations:** 1 Department of Pharmaceutical Sciences, School of Pharmacy, University of Maryland, Baltimore, USA; 2 Center for integrative Medicine, School of Medicine, University of Maryland Baltimore, USA

## Abstract

**Background:**

Dysfunctions in the serotonergic system have been implicated in several neurological disorders such as depression. Elderly individuals who have been diagnosed with clinical depression show elevated cases of neurodegenerative diseases. This has led to suggestions that modulating the serotonin (5-HT) system could provide an alternative method to current therapies for alleviating these pathologies. The neuroprotective effects of bilobalide *in vitro *have been documented. We aim to determine whether bilobalide affects the 5-HT system in the nematode *C. elegans*. The wild type worms, as well as well-characterized 5-HT mutants, were fed with bilobalide in a range of concentrations, and several 5-HT controlled behaviors were tested.

**Results:**

We observed that bilobalide significantly inhibited 5-HT-controlled egg-laying behavior in a dose-dependent manner, which was blocked in the 5-HT receptor mutants (*ser-4, mod-1*), but not in the 5-HT transporter (*mod-5*) or synthesis (*tph-1*) mutants. Bilobalide also potentiated a 5-HT-controlled, experience-dependent locomotory behavior, termed the enhanced slowing response in the wild type animals. However, this effect was fully blocked in 5-HT receptor *mod-1 *and dopamine defective *cat-2 *mutants, but only partially blocked in *ser-4 *mutants. We also demonstrated that acetylcholine transmission was inhibited in a transgenic *C. elegans *strain that constitutively expresses Aβ, and bilobalide did not significantly affect this inhibition.

**Conclusion:**

These results suggest that bilobalide may modulate specific 5-HT receptor subtypes, which involves interplay with dopamine transmission. Additional studies for the function of bilobalide in neurotransmitter systems could aid in our understanding of its neuroprotective properties.

## Background

Serotonin (5-HT) modulates several behaviors in both vertebrate and invertebrate systems and plays an important role in neuronal plasticity and survival. It has also been associated with behavioral deficiencies seen in Alzheimer's patients [1]. The serotonergic system is particularly interesting because of its interactions with many other neurotransmitters systems, such as glutamate, acetylcholine and GABA [2]. In AD, there are drastic decreases in excitatory neurotransmitters, whilst the inhibitory effects of 5-HT on these systems remains relatively stable [3]. These findings have led to suggestions that antagonizing 5-HT receptors, specifically the 5-HT_1 _receptor subtype, could provide an alternative or an adjunct, to current AD therapies. Previous research has found that induced cholinergic and glutamatergic dysfunction [4] were both alleviated by treatment with 5-HT_1A _antagonist WAY 100 635. Experimental evidence also supports a role for 5-HT receptors in learning and memory [2]. Administration of selective serotonin reuptake inhibitors (SSRIs) was reported to stimulate hippocampal neurogenesis in adult rats; and this increase is modulated through different 5-HT receptor subtypes [5]. These studies strongly support physiological, pathophysiological as well as therapeutic roles linking the serotonergic system with cognitive processes.

In the process of identifying behavioral phenotypes of a neuronal amyloid β (Aβ) expressing *Caenorhabditis elegans *strain, we discovered a change of sensitivity to serotonin (5-hydroxytryptamine 5-HT), which was restored by feeding the worms with the *Ginkgo biloba *extract EGb 761 and some of it's constituents [6]. This result led us to hypothesize that certain pharmacologically active constituents of the extract are responsible for protecting the worms against Aβ toxicity; partially through modulation of 5-HT transmission.

Bilobalide, a sesquiterpene trilactone that accounts for approximately 3% of EGb 761 has been shown in many cases to be neuroprotective; particularly in models of acute neurodegeneration and Parkinson's disease. It has been reported that bilobalide protects against glutamatergic excitotoxicity both *in vitro *and *in vivo *[7-9] by antagonizing GABA receptors [10]. Bilobalide has also been shown to inhibit *N*-methyl-D-aspartate-induced activation of phospholipase A_2 _and its resultant phospholipid breakdown [7]. In combination with its inhibition of glutamate-induced death on rat cerebellar granule neurons, this has led to suggestions that bilobalide may be useful in the treatment of certain neurological disorders. In addition to these results, a high dose of bilobalide (100 μM) was shown to reduce the release of excitatory neurotransmitters [11]. In the present study, we seek to test the hypothesis that modulation of the 5-HT system by bilobalide may attribute to its neuroprotective properties.

In *C. elegans*, several behaviors are controlled by the 5-HT system [12,13]. Similar to mammals, there are two major classes of 5-HT receptors in *C. elegans*. Table 1 provides a summation of the different *C. elegans *mutants and their specific mechanisms and/or functions that were utilized in this study. Comparisons are made between the *C. elegans *mutants and their mammalian homologues. As illustrated in table 1, the *mod-1 *receptor encodes an ionotropic 5HT receptor [14]. *Ser-1, ser-4*, and *ser-7 *are members of the metabotropic G-protein-coupled 5HT receptor superfamily [15-17]. Using the available 5-HT transmission mutants (Table 1) and 5-HT controlled behavioral assays, possible genetic targets of bilobalide were demonstrated in the present study.

**Table 1:** C. elegans 5-HT mutants with respect to mammalian counterparts

***C. elegans *5-HT mutants**	**Mammalian homologues**	**Gene product mechanisms**	**Function in *C. elegans***
SER-4	5-HT1 receptors	↓ levels of cAMP	Required for normal inhibition of movement by 5-HT and stimulation of egg-laying by imipramine
SER-1	5-HT2 receptors	↑ IP3, DAG, Ca2+	Required for the stimulation of egg-laying by 5-HT and weakly for pharyngeal pumping
MOD-5	SLC6A4	Na/Cl 5-HT transporter	Required for stimulation of egg-laying in by 5-HT
TPH-1	Tph1	Tryptophan hydroxylase	
MOD-1	GABAB3	5-HT-gated Cl channels	Required for enhanced slowing response exhibited by food-deprived animals upon encountering a bacterial food source

## Results

### 1. Inhibition of 5-HT stimulated egg-laying behavior by bilobalide requires serotonin receptors *mod-1 *and *ser-4*

**Figure 1:**
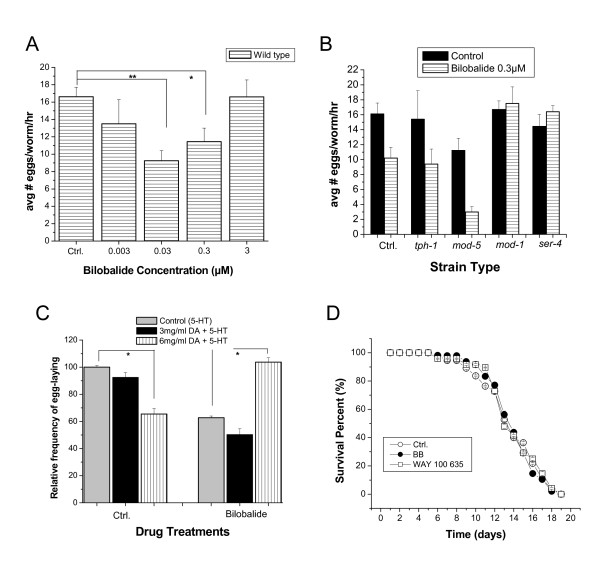
**Serotonin stimulated egg-laying and survival assay**. **(A) **Wild type young larvae (L1) were fed with bilobalide (BB) ranging from 3 nM to 3 μM for 4 days. The number of eggs was scored 60 minutes after 5-HT (5 mg/ml) was added in 96-well plates (*p < 0.05, **p < 0.001). **(B) **Egg-laying response to BB in wild type and 5-HT mutants adult day 1 worms. **(C) **Wild type adult day 1 worms (8 from each group) were fed with Bilobalide (BB) (0.3 μM) from L1 stage. Animals were then pre-incubated with dopamine for 2 h before being placed in a 5-HT solution. The bars represent the mean of the number of eggs from three individual assays; *p < 0.05. **(D) **Life span assay in N2 *C. elegans *fed either Bilobalide (BB) 0.3 μM (closed circle, bilobalide), WAY 100 635 (open squares, WAY 100 635) 0.3 μM or the vehicle control (open circle, Ctrl). Worms were transferred for 4 consecutive days during their egg-laying phase, and then every other day after the egg-laying phase was completed. Data are expressed as the percentage (%) of survival in an age-synchronized population. Three independent experiments were performed with number of worms totaling 200.

Egg-laying is one of several well-defined behaviors *C. elegans *that are controlled by 5-HT. Exogenous levels of 5-HT can stimulate egg-laying behavior, a response extremely useful in identifying the action of drugs on specific neurotransmitter pathways [18,19]. To determine if bilobalide alters 5-HT stimulated egg-laying, the wild type worms (adult day 1, 30 each group) were fed with bilobalide (BB) from L1 young larvae, followed by the egg-laying assay. When several concentrations of BB ranging from 0.003 to 30 μM was administered to the worms, a dose-dependent inhibition by BB was statistically significant at concentrations of 0.03 and 0.3 μM (n = 3, p < 0.05 Fig 1A) suggesting a specificity of this inhibition. However, concentrations higher than 3 μM were not statistically different from the control (data not shown).

**Figure 2:**
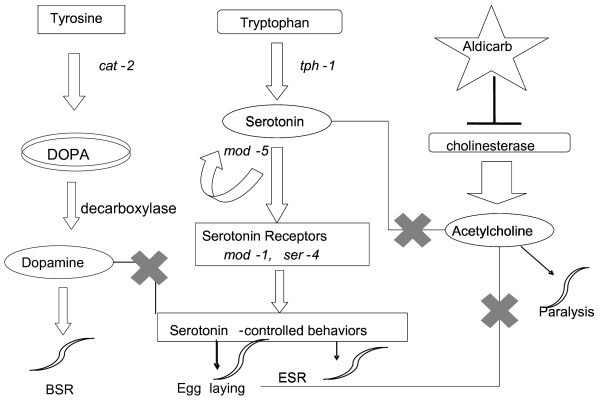
**Schematic diagram of the neurotransmitter controlled behaviors and the associated mutant used in this study**. The CAT-2 gene encodes for tyrosine hydroxylase and is the primary pathway for dopamine (DA) synthesis. Serotonin synthesis requires the TPH-1 tryptophan hydroxylase gene. Serotonin is removed from the synaptic space by the MOD-5 serotonin reuptake transporter. MOD-1 receptor encodes an ionotropic 5HT receptor and SER-4 belongs to the class of metabotropic G-protein-coupled 5HT receptors, and is homologous to mammalian 5-HT1 receptor class. The drug aldicarb is a cholinesterase inhibitor that causes an accumulation of acetylcholine at the neuromuscular junction, which eventually leads to paralysis. Exogenous 5-HT can inhibit this behavior. 5-HT stimulates egg-laying in *C. elegans *and both dopamine and acetylcholine inhibit it.

We then sought to identify where BB might disrupt serotonergic neurotransmission. The *tph-1 *mutant has a deletion in the 5-HT biosynthetic enzyme, tryptophan hydroxylase. This mutant lacks any detectable 5-HT and accumulates an excess amount of fertilized eggs [20], although its egg-laying circuitry remains responsive to exogenous 5-HT. The *mod-5 *mutant (Fig. 2) encodes for the only serotonin reuptake transporter (SERT) that has been discovered in *C. elegans *[21]. *mod-1 *encodes an ionotropic 5-HT receptor (5-HTR) [14] and SER-4 is a member of metabotropic G-protein coupled receptors, similar to mammalian 5-HT_2 _and 5-HT_1A _subtypes [16]. Both of these receptor subtypes are expressed in the neurons. When 5-HT stimulated egg-laying was scored in wild type and four 5-HT system defective mutants *(tph-1, mod-5, mod-1 *and *ser-4*) treated with BB, the inhibitory effect by BB was blocked in *mod-1 *and *ser-4 *mutants. The *tph-1 *and *mod-5 *mutants respond to BB in a similar manner as wild type (Fig. 1B), which suggest that the presynaptic component may not be required for the effect of BB on this particular behavior. These results indicate that the 5-HT receptors (*mod-1 and ser-4*) (Fig. 2) may be necessary for the BB effect in the egg-laying assay.

Since BB displayed inhibitory effects on 5-HT stimulated egg-laying, we next sought to determine if it would interact with a known inhibitor of egg-laying in *C. elegans*, the neurotransmitter dopamine (DA) [19]. Various concentrations of BB failed to inhibit egg-laying in the *cat-2 *DA mutant (data not shown). In wild type animals that were pre-incubated with increasing concentrations of exogenous DA, there was a dose-dependent inhibition of their egg-laying behavior (Fig. 1C). Wild type worms that were fed BB (0.3 μM) and then pre-treated with DA for 2 h before 5-HT stimulated egg-laying assay did not show an inhibition in egg-laying by DA (Fig. 1C). In fact, the egg-laying response was similar to the response seen in the wild type worms with no treatment at all (Ctrl. 100% ± 1.1, BB 62.7% ± 1.3 p < 0.05) (DA 3 mg/ml 92% ± 3.5, BB 50.3% ± 4.2) (DA 6 mg/ml 65.4% ± 4.1, BB 103% ± 3.2 p < 0.05).

Since bilobalide had a significant effect on 5-HT-controlled egg-laying, we wanted to determine whether it, or a known 5-HT receptor antagonist WAY 100 635 (0.3 μM), would have any impact on life span and to ensure that the results seen with BB treatment were not a consequence of toxicity. Neither BB (0.3 μM) nor WAY 100 635 significantly affected the life span of wild type worms (Fig 1D, BB: 13.76 ± 0.54; WAY 100 635:13.97 ± 0.6; control group: 13.8 ± 0.31). This result excludes the possibility of a general effect by bilobalide on the life span of *C. elegans*.

### 2. Bilobalide potentiates a 5HT controlled experience-dependent locomotory behavior

**Figure 3:**
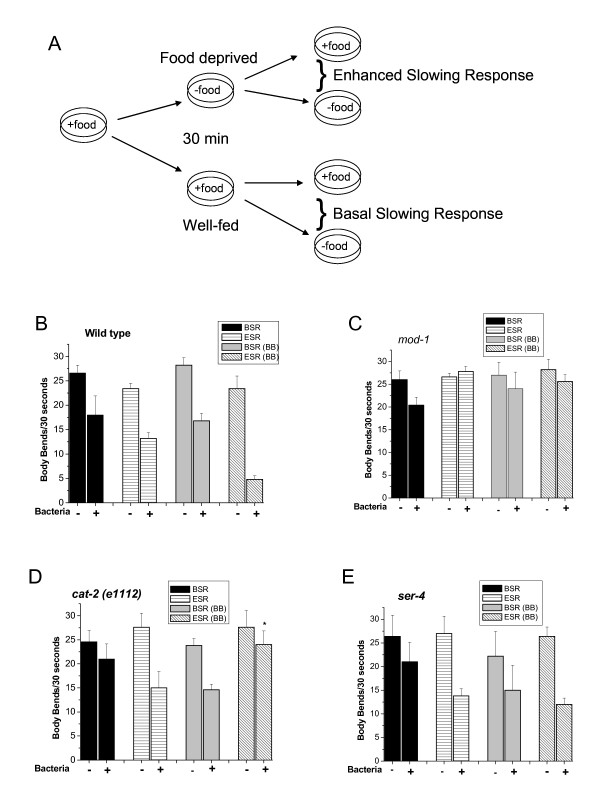
**Serotonin-controlled learning behavioral**. **A**. Schematic diagram of Slowing Responses in *C. elegans*. The basal and enhanced slowing responses (BSR), which are mediated by dopamine and serotonin, respectively are used to associate previous feeding experience. For the basal slowing response worms are well fed up until they are assayed. For the enhanced slowing response (ESR), there is a 30 min starvation period before they are assayed. **(B) **Basal (BSR) and enhanced (ESR) slowing responses in wild type animals fed with control or bilobalide (BB). **(C) **BSR and ESR in the *mod-1 *mutant worms fed with vehicle control or treated with BB. **(D) **BSR and ESR in the *cat-2 *mutant worms control or treated with BB. Four separate trials were performed for each strain. Error Bars represent the standard error; asterisk; p < 0.05, Student's *t *test. **(E) **BSR and ESR in the *ser-4 *mutant worms fed with vehicle control or with BB.

**Figure 4:**
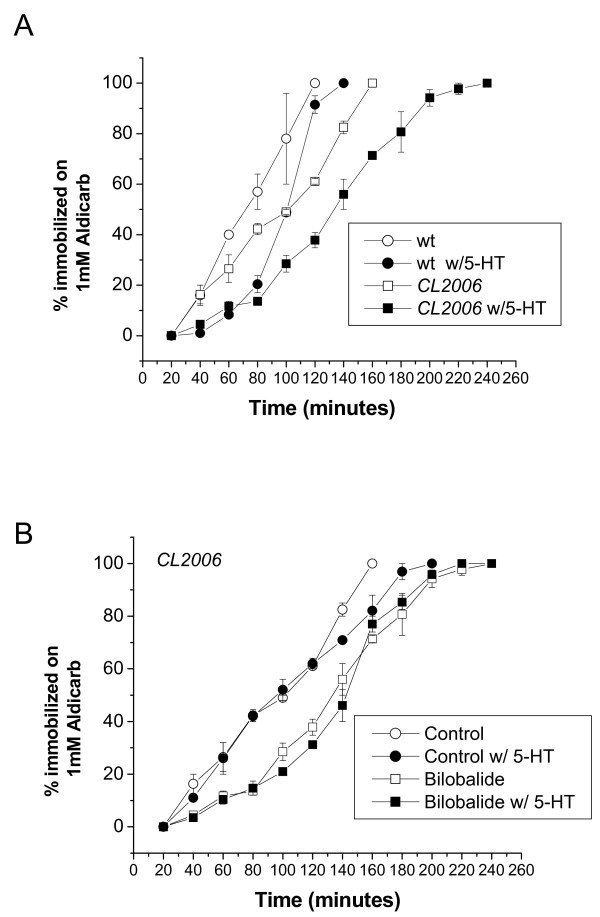
**Aldicarb Sensitivity Assay**. **(A) **Wild type (wt) or worms that constitutively express human amyloid beta in the muscle cells (CL2006) were pre-incubated for 2 hours on plates with or without 2 mM of 5-HT. Aβ worms (CL2006), exhibit a natural resistance to aldicarb, in comparison to wild type controls. Each group contained 30 animals that were placed on 1 mM of aldicarb and scored until death. **(B) **Aβ worms (CL2006) were fed BB (0.3 μM) then pre-treated with 2 mM of exogenous 5-HT for 2 hrs. BB treatment has no effect on aldicarb sensitivity in transgenic Aβ worms (CL2006) pre-exposed to 5-HT. Data was obtained from three independent experiments. Each group consisted of 30 worms.

We decided to utilize the basal and enhanced slowing responses (BSR and ESR, illustrated in Fig. 3A), which are measures of behavioral plasticity in the worms, to determine the effect of BB on an experience-dependant behavior. The slowing responses are used to associate prior feeding experience with response to food. This makes these responses a type of associative learning behavior (Fig. 3A). In the worms, the basal and enhanced slowing responses are mediated by dopamine and serotonin, respectively. Sawin et al. demonstrated that *mod-1 *mutant worms were defective in the 5-HT controlled enhanced slowing response. This group also reported that *cat-2 (e1112) *mutants that are deficient in the enzyme tyrosine hydroxylase, which is required for the synthesis of dopamine (DA), have normal serotonergic transmission but are defective in the DA mediated basal slowing response [12]. This same study also showed that when these animals are pre-incubated with exogenous DA, the basal slowing response is rescued. In the present study, we show that feeding the worms 0.3 μM of BB for three days before the assay significantly potentiated the ESR in wild type worms (Fig. 3B). However, this enhancement by BB was completely blocked in the *mod-1 *(Fig. 3C) and *cat-2 (e1112) *(Fig. 4C), p = 0.04, but not in *ser-4 *(Fig. 3E), mutant animals. Previous studies have reported that deficiencies in the serotonin receptors could lead to defects in the enhanced slowing response [14]; although a *mod-5 *mutation leads to a hyper-enhanced slowing response [21]. In the presents study, the *ser-4 *mutant animals did not show any abnormalities in the ESR, although there were slight changes in the BSR for both *ser-4 *and *mod-1 *(Fig. 4C&E). These results suggest that another mechanism for the neuroprotective effects of BB may involve altering the serotonergic as well as the dopaminergic system.

### 3. Animals that constitutively express Aβ show reduced aldicarb sensitivity

In the presence of the acetylcholinesterase inhibitor aldicarb, accumulation of acetylcholine (ACh) in the synapse causes the worms to paralyze; which is inhibited by 5-HT [22]. This assay was adapted to determine the impact of Aβ-expression on the interaction between 5-HT and ACh. Wild type worms (Fig 4A. open circles) were exposed to 1 mM aldicarb and scored for progressive paralyses every 20 min. In Fig. 4A (filled circles), animals that were pre-incubated with 5-HT show a decrease in aldicarb sensitivity. Next, we compared the aldicarb sensitivity phenotype in wild type (N2) and a *C. elegans *mutant that constitutively expresses human amyloid beta in the muscle cells (CL2006). The results show that Aβ mutants (Fig. 4A, open squares, CL2006) were more resistant to aldicarb exposure than the wild type worms (open circles, N2, Fig. 4A) indicating a defect in ACh transmission in the Aβ strain. When Aβ mutants were pre-incubated with 5-HT (filled squares, CL2006 w/5-HT, Fig. 4A), their paralyses were remarkably delayed compared with wild type worms pre-incubated with 5-HT (filled circles N2 w/5-HT Fig. 4A) or the Aβ mutants that were not pre-incubated with 5-HT (open squares, CL2006). These results extend our previous findings [6] and suggest that Aβ expression alters both 5-HT as well as ACh transmission in *C. elegans*.

We next wanted to elucidate whether BB modulates Aβ-reduced aldicarb sensitivity. The mutant worms were fed BB (0.3 μM) for 4 days and then pre-incubated for 2 hrs on 1 mM of 5-HT followed by the aldicarb paralysis assay. Interestingly, the Aβ worms fed with BB (0.3 μM open squares, control Fig. 4B) did not rescue the impairment compared with non-treated controls (filled squares, control (Fig. 4B), indicating that BB is not specific to Ach transmission as well as its associated effect by Aβ expression.

## Discussion

Serotonin has been shown to modulate many different behaviors in the nematode *C. elegans*. In the present study, we demonstrate a novel mechanism by which bilobalide (BB) modulates behavioral plasticity in *C. elegans *by antagonistically regulating serotonergic receptors. BB antagonizes egg-laying behavior via modulation of ionotropic MOD-1 and metabotropic SER-4 receptors in *C. elegans*, which are homologues to mammalian GABA-gated chloride channels and 5-HT_1A_/5-HT_2A _receptors, respectively. In addition, we demonstrated that Aβ expression in the worms reduced their sensitivity to aldicarb, an acetylcholinesterase inhibitor (AChEI). However, BB did not affect this behavior. Our results suggest that modulation of the 5-HT system by BB partially underlies its neuroprotective and efficacious effects in several *in vitro *systems [8-11]. Recent studies revealed a role of 5-HT receptors (5-HTR) in controlling *C. elegans *life span [23]. A mutation in the *ser-1 *gene, which encodes a 5-HT2-like receptor [16], dramatically increased life span (by 46%) and significantly enhanced thermotolerance and UV resistance in the worms [13]. These finding may provide an explanation for our previous data that EGb 761 extends life span [24]; an effect that could be partially due to bilobalide modulation of the 5-HT system. Supporting this view, another independent study that screened 88,000 compounds for life span extension in *C. elegans *found that the most potent life span extending compounds are antidepressant drugs that are antagonists of 5HT2_A _receptors in humans [25]. Despite unfavorable outcome from clinical studies of EGb 761[26], the unique features of its constituents warrant future investigation [6].

The egg-laying assay in *C. elegans *has long served as a behavioral marker for serotonergic neurotransmission. In the present study, application of exogenous 5-HT stimulated egg-laying in wild type *C. elegans *[27], which was significantly inhibited in worms fed BB (Fig 1A). In *tph-1 *mutants, which are deficient in tryptophan hydroxylase, an enzyme essential for synthesis of 5-HT [20], BB was still able to inhibit 5-HT stimulated egg-laying. The inhibition of egg-laying behavior by BB was blocked in *mod-1 *and *ser-4*, two 5-HTR mutants (Fig 1B), suggesting that these receptors are required for BB activity in *C. elegans *5-HT-stimulated egg-laying behavior. Another possibility is that BB inadvertently affects 5-HTR by effecting dopamine (DA) function. Extensive research performed in rats, has demonstrated that DA can be enhanced or diminished by compounds that interact with specific 5-HTR subtypes [19]. It has been reported that a mutation in MOD-1, which is a 5-HT-gated ion channel similar to mammalian GABA-Glycine-chloride channels, blocks DA inhibition of egg-laying in *C. elegans*. Previous studies have demonstrated that BB antagonizes GABA and glycine receptors in embryonic cortical slices [28] and it binds GABA_A _receptors at therapeutically relevant concentrations [10]. Antagonizing 5-HT receptor SER-4 by BB in *C. elegans *implicates possible beneficial effects in learning and memory. 5-HT_1A _receptor in mammals is structurally homologues to the SER-4 receptors in *C. elegans*. In the mammalian system, activation of 5-HT_1A _receptors causes defects in learning and memory. On the other hand, activation of the 5-HT_2A _and 5-HT_2C _receptors facilitates the formation of memory [2].

Although exogenous 5-HT stimulates egg-laying, it has an inhibitory effect on locomotion [14]. 5-HT reduces locomotion rates by inhibiting the release of ACh at the *C. elegans *neuromuscular junction [22]. This in turn, leads to resistance to the cholinesterase inhibitor aldicarb, shown as reduced paralysis in the worms exposed to aldicarb. For the first time, we observed that a transgenic *C. elegans *strain that constitutively expresses human Aβ in the muscle cells (CL2006) was resistant to aldicarb (Fig. 4A). Previous reports demonstrated that worms treated with 5-HT receptor antagonist ketanserin and methiothepin became hypersensitive to aldicarb, whereas DA receptor antagonist had no effect [22]. It should be noted that modulation of 5-HT receptors in mammals is subtype and synaptic localization dependent. Activation of 5-HT_1A _heteroreceptors resulted in a decrease in ACh release [29] and impairment of cognitive functions [30], whereas, activation 5-HT_1A _autoreceptors can enhance cognition [31].

The restoration of the basal slowing response by BB in the *cat-2 *mutants was surprising. Although the *cat-2 *mutants are deficient in tyrosine hydroxylase, the enzyme needed for DA synthesis, it has been shown that DA synthesis can occur through alternative routes and that these worms still have some functioning DA [32], although significantly less than wild type. These results suggest that BB can modulate the interplay between the serotonergic and dopaminergic systems, which is believed to a basic mechanism for synaptic plasticity in the worms [33]. Antagonist of 5-HTR subtypes appear to enhance activation and signaling of neurotransmitter systems that are known to be involved in cognition [34].

It is important to separate the effects of 5-HT on egg-laying and locomotion [35,36]. While application of exogenous 5-HT stimulates egg-laying [27] it inhibits locomotion [14]. In *C. elegans *egg-laying circuitry, there are at least three separate responses to 5-HT [37]. Potential interactions of different receptors subtypes [38] could also account for the effects that are seen with BB. MOD-1 and SER-4 have been shown to provide inhibitory input into the egg-laying process [19,37] and *ser-4; mod-1 *double mutants actually lay more eggs than wild type animals in the 5-HT stimulated egg-laying assay [38]. It is also important to note that stimulation of egg-laying by 5-HT occurs via receptors expressed in the muscles and inhibition occurs via receptors located in the neurons [37]. Since many of the mechanisms of BB remain unclear, it is possible that its modulation of these behaviors is location specific. So, inhibition of egg-laying behavior and the potentiation of the locomotory behaviors are not necessarily contradictory.

Several studies have demonstrated that antagonism of 5-HT_1A _receptors is beneficial in cognitive processes. WAY 100635 and NAD-299 (robalzotan) two selective 5-HT_1A _receptor antagonist, were able to attenuate the impairments in passive avoidance behavior in rats elicited by scopolamine [34]. Also, a new 5-HT_1A _receptor antagonist, WAY 101405 was able to reverse cognitive impairments caused by scopolamine, in both the novel object recognition and contextual fear condition paradigms in rats [39]. Lecozotan, a 5-HT_1A _antagonist that has completed phase I clinical trials, was shown to potentiate the stimulated release of glutamate and acetylcholine in the hippocampus [40]. Although we demonstrated in this study that BB can interact with both serotonergic and dopaminergic signaling in *C. elegans*, its role may be more important in neuroprotection than disease alleviation since there were no observed effects on Aβ toxicity.

In conclusion, bilobalide modulates interplay of 5-HT and dopamine neurotransmission systems. Additional studies for the function of bilobalide in neurotransmitter systems would further our understanding of its neuroprotective properties.

## Methods

### Materials

Bilobalide (BB), serotonin creatinine sulfate complex (5-HT), dopamine hydrochloride (DA) and aldicarb were all purchased from Sigma (St. Louis, MO). Stock solutions of all chemicals were made either with 95% ethanol, M9 Buffer or distilled water. The final concentration of ethanol, when dissolved in the food (*Escherichia coli *strain OP50) did not exceed 0.01%. All chemicals/drugs for treatment of experimental animals were added directly to the OP50 food source.

### *C. elegans *strains

*mod-1 (ok103), ser-4*(*C. elegans *Gene Knockout Consortium), *tph-1 (mg280), mod-5(n3314), cat-2(e1112) *and N2 (Bristol) were all obtained from the *Caenorhabditis *Genetics Center, University of Minnesota. The transgenic strain CL2006 has the *unc-54/Aβ*_1–42 _and *rol-6 *(*su1066*) transgenes integrated into the worm's chromosome and it constituently expresses Aβ_1–42 _in the muscle cells [41]. CL2006 was a gift from Dr. CD Link from the University of Colorado-Boulder. All animals were grown on solid nematode growth media (NGM), with 100 μl OP50 containing either vehicles or chemicals, at 20°C from egg or L1 (day 1 young larvae) until assayed, unless stated otherwise.

### *C. elegans *maintenance and treatment

All animals were cultured at 20°C under standard laboratory conditions. To age synchronize animals, adult nematodes were transferred to fresh NGM plates and allowed to lay eggs for 2–4 h. Isolated hatchlings from the synchronized day 1 young larvae (L1), were cultured on fresh NGM plates in a 20°C temperature-controlled incubator (model 2005; Sheldon Manufacturing, Cornelius, OR). The worms were fed the drugs either from stage L1 (1 d of age) or starting from the egg.

### Behavioral Studies

#### Egg-laying Assay

The average number of eggs released per hour was quantified according to a well established assay [18,19,27], except animals were assayed ~40 ± 2 h after the L4 young larvae stage, so that animals could be fed the drugs for a full three days. Age-synchronized well-fed L4 young larvae were transferred to fresh plates seeded with OP50 containing either vehicle or Bilobalide and allowed to develop for ~20 ± 2 h at 20°C. After another 20 h, the resultant young adults were assayed for their response to the drugs. Individual young adults were transferred to a 96-well plate containing 100 μl of a 5 mg/ml solution of serotonin creatinine sulfate complex dissolved in M9. The number of eggs released at room temperature was scored after 60 min.

#### Life span Assay

The life span assay was performed as described previously [42]. Age synchronized animals were fed with 0.3 μM of Bilobalide from L4 and maintained at 20°C. Animals were transferred daily for 4 consecutive days until they ceased to lay eggs to avoid overlapping generations. Worms were scored as dead if they did not respond to a touch stimulus by a platinum loop.

#### Aldicarb Sensitivity Assay

The acetylcholinesterase inhibitor aldicarb, results in a toxic accumulation of acetylcholine (ACh) in the neuromuscular junction. Aldicarb sensitivity was assayed in adult day 1 animals by analyzing the onset of paralysis following treatment with 1 mM aldicarb. Animals were prodded every 20 min with a platinum wire loop following exposure to drug. As indicated, some worms were pre-incubated with 5-HT for 2 h prior to the assay. Worms were non-treated or fed with BB (0.3 μM), starting from the egg.

#### Locomotory Rate Assays

Assays were performed according to Sawin et al (Sawin, 2000) with slight modifications. A ring of *E. coli *strain OP50 was placed on a 5 cm Petri plate containing NGM. Assay plates were freshly spread with bacteria and allowed to incubate at room temperature overnight. Age synchronized young adult hermaphrodites (~20 ± 2 hrs after L4 larval stage) were tested. For the well-fed animals, we measured the locomotory rate by removing 5 animals from bacteria-containing plates and washing the animals three times in S basal buffer, and then quickly transferring them to an assay plate. Any buffer that remained on the plates was absorbed with a Kimwipe. Five minutes after the worms were transferred; we measured the number of body bends in 30 second intervals for each of the 5 animals that were on the assay plate. The angle of a body bend is typically more than 90°.

For the starved animals, 5–10 animals were washed free of bacteria in S basal buffer (3X's) and then transferred to a 5 cm NGM agar plate that was free of bacteria. Excess buffer was removed with a Kimwipe. The animals were incubated on these plates for 30 minutes at room temperature. After 30 minutes of starvation, five worms were transferred to assay plates and the locomotory rate was measured as described above.

#### Neurotransmitter Pre-treatment

Solutions of dopamine hydrochloride (50 mM) were prepared in M9 buffer and 400 μl of the solution or M9 was added to each 5 cm plate containing ~10 ml of agar and a bacterial lawn so that the final concentration of DA solution would be ~2 mM. The plates were allowed to dry at 1 hr at room temperature and then 40–50 worms (treated and untreated were transferred to each plate. Animals were incubated for 2 hrs at 20°C and then assayed according to the locomotory assay mentioned above [12].

#### Data Analysis

Data are analyzed for statistical significance by independent Student's *t *test of untreated and drug-treated animal groups using Origin 6.0 software (Microcal Software, Northampton, MA). In all cases a value of *p *< 0.05 indicates a significant difference.

## Abbreviations used

AD: Alzheimer's disease; BB: bilobalide; 5-HT: serotonin; DA: dopamine; 5-HTR: serotonin receptors; Aβ: amyloid beta peptide; BSR: basal slowing response; ESR: enhanced slowing response.

## Authors' contributions

MKB carried out all behavioral assays, statistical analysis and drafted the manuscript. YL participated in general design of the experiments and edited the final version of the manuscript. All authors have read and approved the final manuscript.
